# Clinicopathologic characteristics and prognosis for male breast cancer compared to female breast cancer

**DOI:** 10.1038/s41598-021-04342-0

**Published:** 2022-01-07

**Authors:** Nan Yao, Wenzai Shi, Tong Liu, Sarah Tan Siyin, Weiqi Wang, Ning Duan, Guoshuai Xu, Jun Qu

**Affiliations:** 1 Department of General Surgery, Aerospace Center Hospital, Beijing, 100089 China; 2 Department of Hepatobiliary Surgery, Peking University International Hospital, Beijing, 100038 China; 3 Department of Gastrointestinal Surgery, Beijing Shijitan Hospital, Beijing, 100038 China; 4 Department of General Surgery, Beijing Children’s Hospital, Beijing, 100038 China; 5 Aerospace Center Hospital, Yuquan Road 15, Haidian District, Beijing, 100038 China

**Keywords:** Cancer, Diseases, Oncology

## Abstract

Male breast cancer (MBC) is rare. Due to limited information, MBC has always been understudied. We conducted a retrospective population-based cohort study using data from the National Cancer Institute’s Surveillance, Epidemiology, and End Results (SEER) program. The clinical and biological features of female breast cancer (FBC) patients were compared with MBC patients. Cox regression models and competing risks analyses were used to identify risk factors associated with cancer-related survival in MBC and FBC groups. Results showed that MBC patients suffered from higher TNM stages, tumor grades, and a higher percentage of hormone receptor-positive tumors, compared with FBC patients (all *p* < 0.05). In addition, the breast tumor locations varied a lot between males and females (*p* < 0.05). FBC patients were associated with superior overall survival than MBC patients. Results from multivariate cox regression and competing risks analyses showed age, race, T, N, M-stages, tumor grades, estrogen receptor (ER)/progesterone receptor (PR) and human epidermal growth factor receptor-2 (HER-2) overexpression were independent prognosis factors in FBC patients (all *p* < 0.05). MBC patients had similar risk factors to FBC patients, but PR and HER-2 status did not independently influence survival (all *p* > 0.05). Tumor location was an independent prognostic factor for both gender groups.

## Introduction

Breast cancer is one of the most common malignant tumors, and the leading cause of cancer-related death in women worldwide. In 2018, there were an estimated 2.1 million new cases of breast cancer and 627,000 deaths from breast cancer worldwide^1^. Though it is rare, breast cancer in men accounts for 1% of all breast cancer cases^2,3^.

Given the low incidence, previous studies on male breast cancer (MBC) have suffered from small sample sizes, short follow-up time, limiting their interpretability. And the therapeutic strategies for MBC patients are commonly extrapolated from those used to treat postmenopausal female breast cancer (FBC) patients^4,5^. No existing evidence-based data supports this female-to-male extrapolation. Literature has suggested that MBC has biological differences compared with FBC. MBC patients are typically associated with advanced stages, higher grades, higher prevalence of hormone receptor-positive, and a worse prognosis^6–11^. Furthermore, studies have proposed that MBC patients are insensitive to adjuvant therapy, and an underutilization of therapy in MBC patients compared with FBC patients^12,13^. Therefore, it may be inappropriate to adopt the clinical applications of female-to-male extrapolation.

In the current study, we attempt to compare the clinicopathologic characteristics and prognosis between MBC patients and FBC patients by drawing data from the National Cancer Institute’s Surveillance, Epidemiology, and End Results (SEER) Database from the beginning of 2010 to the end of 2014, with the aim of better understanding gender differences and specificity of MBC.

## Materials and methods

This is a retrospective cohort study of breast cancer patients diagnosed in the SEER database 8.3.4 from the beginning of 2010 to the end of 2014. SEER collects cancer incidence data from population-based cancer registries covering approximately 34.6% of the U.S. population. It also records data of patients’ clinicopathological characteristics, and vital status during follow-up. Breast cancer cases were identified according to the 3rd edition of the International Classification of Diseases for Oncology (ICD-O-3).

**Figure 1:**
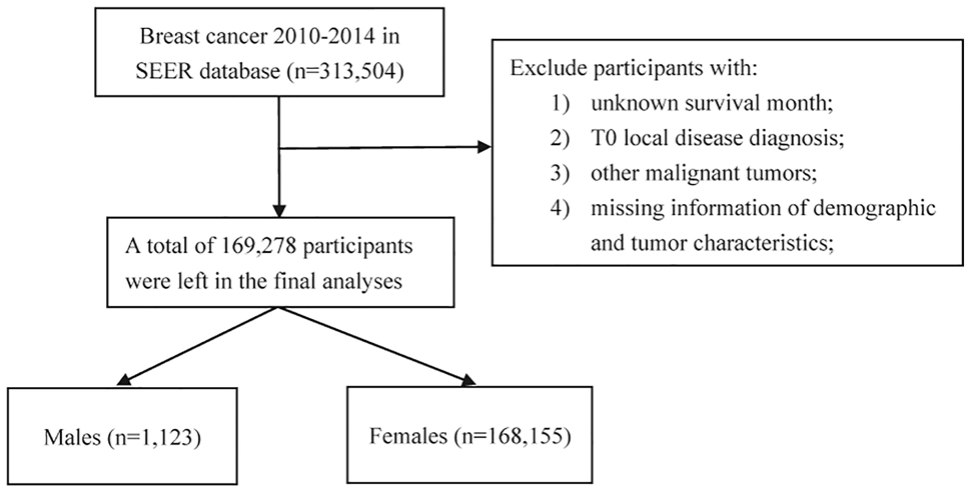
Flow chart of patients’ selection for final analysis.

A total of 313,504 patients with breast cancer were identified in the SEER database. Patients were excluded if they had 1) survival month that was 0 or unknown; 2) T0 local disease diagnosis; 3) other malignant tumors. 4) missing information for demographic and tumor characteristics including sex, age, laterality (left, right, bilateral), tumor location, race (white, black, and other), pathological type (ductal, lobular, and other), TNM stage, histological grade (well-differentiated, moderately differentiated, poorly-differentiated and undifferentiated), surgical treatment of breast cancer, estrogen receptor (ER), progesterone receptor (PR), and HER-2. A total of 169,278 patients (1,123 males and 168,155 females) remained in the final analysis (Fig. 1). Surgical treatment of breast cancer was defined as patients who received any type of surgical resection of the primary tumor. Histology was classified into three subtypes: ductal, lobular and others including mucinous adenocarcinoma, non-small cell carcinoma, adenocarcinoma and other rare types of cancer. All patients have given prior informed consent to being registered in SEER database.

### Statistical analysis

All statistical analyses were performed using SAS statistical software (version 9.4). The characteristics of the subjects with normal distribution were expressed as mean ± standard deviation and compared using t test. Categorical variables were represented as absolute value with percentage and the Chi-square test was used for comparison between male and female patients. Kaplan–Meier survival curves were generated to compare differences in survival probabilities over time between groups, and the equality of these curves was tested using a log-rank statistic.

The interval from the date of cancer diagnosis to the endpoint was calculated as survival time (in months). The endpoint was defined as one of the three events, whichever occurred first: date of breast cancer-related death, date of non-breast cancer related death, or the date used as the cutoff for the study. Cox regression models were generated to describe the relationship between clinicopathologic features and risk of breast cancer-related death among MBC patients and FBC patients.

Non-breast cancer related death may occur before the occurrence of breast cancer-related death during the follow-up period which hinders us from identifying the existence of breast cancer cases. The traditional multivariate COX regression model may markedly overestimate the risk of breast cancer^14^. To avoid overestimation and to improve accuracy, the cause-specific hazard model (CS model) and sub-distribution hazard function model (SD model) were used to calculate the absolute risk of breast cancer-related death. A two-sided *p*-value < 0.05 was considered statistically significant in this study.

### Ethics approval and consent to participate

The study was approved by the Ethics Committee of Aerospace Center Hospital and was complied with the Declaration of Helsinki.

## Results

### Differences in clinical and pathological characteristics between males and females

MBC (n = 1,123/169,278) represented 0.66% of all breast cancers. The common descriptive characteristics of both genders are presented in Table 1. The median age at diagnosis for MBC was 63.45 ± 10.81 years old compared to 58.96 ± 12.14 years old for FBC (*p* < 0.001). In MBC patients, the race of patients was predominantly white (80.23%), with 14.34% black and 5.43% other races. In FBC patients, 79.19% were white, 11.04% were black and 9.77% were of others races (*p* < 0.001). There was no difference in laterality between men and women (*p* = 0.085). MBC patients tend to have larger tumor sizes (*p* < 0.001), a higher percent of lymph node (*p* < 0.001) and organ metastasis (*p* < 0.001) compared with FBC patients. MBC patients were also more likely to present with advanced grades (*p* < 0.001). In terms of pathological types, 84.86% of MBC patients were invasive ductal carcinoma compared with 77.81% in FBC patients. Lobular carcinomas accounted for only 0.62% of breast cancer in MBC patients by the contract of 8.14% in FBC patients (*p* < 0.001). Compared with FBC patients, MBC patients were more likely to express hormone receptor-positive. 97.42% of men and 82.72% of women had ER-positive tumors (*p* < 0.001). Similarly, MBC patients were more likely to have PR-positive tumors than FBC patients (91.63% vs. 72.46%, *p* < 0.001). Compared with FBC patients, MBC patients exhibited a lower percentage of HER-2 overexpression (11.04% vs. 14.68%, *p* < 0.001).

**Table 1:** Disease characteristics of male versus female breast cancer.

	Males	Females	t/X^2^	*p*-value
n	1,123	168,155		
Age (years)	63.45 ± 10.81	58.96 ± 12.14	− 12.34	< 0.001
**Races (%)**			32.73	< 0.001
White	901 (80.23)	133,163 (79.19)		
Black	161 (14.34)	18.557 (11.04)		
Other	61 (5.43)	16,435 (9.77)		
**Laterality (%)**			4.93	0.085
Left	516 (45.95)	82,798 (50.6)		
Right	607 (54.05)	85,344 (48.9)		
Bilateral	0 (0)	13 (0.5)		
**Tumor locations (%)**			4906.95	< 0.001
Upper-inner	48 (4.27)	23,642 (14.06)		
Upper-outer	175 (15.58)	65,854 (39.16)		
Lower-inner	25 (2.23)	10,721 (6.38)		
Lower-outer	58 (5.16)	14,188 (8.44)		
Nipple	69 (6.14)	638 (0.38)		
Central portion	538 (47.91)	8,740 (5.20)		
Axillary tail	1 (0.09)	887 (0.53)		
Overlapping lesion	209 (18.61)	43,485 (25.86)		
**Grades (%)**			87.80	< 0.001
I (Well)	131 (11.67)	38,377 (22.82)		
II (Moderately)	596 (53.07)	73,013 (43.42)		
III (Poorly)	395 (35.17)	56,229 (33.44)		
IV (Undifferentiated)	1 (0.09)	536 (0.32)		
**T stages (%)**			180.73	< 0.001
T1	492 (43.81)	100,663 (59.86)		
T2	513 (45.68)	52,779 (31.39)		
T3	43 (3.83)	9,772 (5.81)		
T4	75 (6.68)	4,941 (2.94)		
**N stages (%)**			78.84	< 0.001
N0	639 (56.90)	115,159 (68.48)		
N1	337 (30.01)	39,239 (23.34)		
N2	101 (8.99)	8,699 (5.17)		
N3	46 (4.10)	5,058 (3.02)		
**M stages (%)**			16.36	< 0.001
M0	1,064 (94.75)	162,878 (96.86)		
M1	59 (5.25)	5,277 (3.14)		
**Histology (%)**			85.16	< 0.001
Ductal	953 (84.86)	130,836 (77.81)		
Lobular	7 (0.62)	13,681 (8.14)		
Others	163 (14.51)	23,638 (14.06)		
**ER (%)**			169.30	< 0.001
Positive	1,094 (97.42)	139,103 (82.72)		
Negative	29 (2.58)	29,052 (17.28)		
**PR (%)**			20,596	< 0.001
Positive	1,029 (91.63)	121,851 (72.46)		
Negative	94 (8.37)	46,304 (27.54)		
**HER-2 (%)**			20.64	< 0.001
Negative	960 (85.49)	139,936 (83.22)		
Equivocal	39 (3.47)	3538 (2.10)		
Positive	124 (11.04)	24,081 (14.68)		
**Surgery (%)**			0.008	0.930
Yes	1069 (95.19)	160,164 (95.25)		
No	54 (4.81)	7991 (4.75)		

*ER* estrogen receptor, *PR* progesterone receptor, *HER-2* human epidermal growth factor receptor 2. The definitions of T, N, M were referred to pathologic stage groups (pTNM).

Among MBC patients, there were 47.91% of tumors located with the central portion, followed by 15.58% in the upper-outer quadrant, 6.14% in the nipple, 5.16% in the lower-outer quadrant, 4.27% in the upper-inner quadrant, 2.23% in the lower-inner quadrant, 0.09% in the axillary tail, and the remaining 18.61% were classified as overlapping lesion. However, in FBC patients, there were only 5.20% of tumors located in the central portion, and tumors primarily located in the upper-outer accounted for 39.16%. The rest were located in the upper-inner quadrant (14.06%), lower-inner quadrant (6.38%), lower-outer quadrant (8.44%), nipple (0.38%), axillary tail (0.53%), and overlapping lesion (25.86%).

### The association of clinicopathologic characteristics with the cancer-related death risk

**Figure 2:**
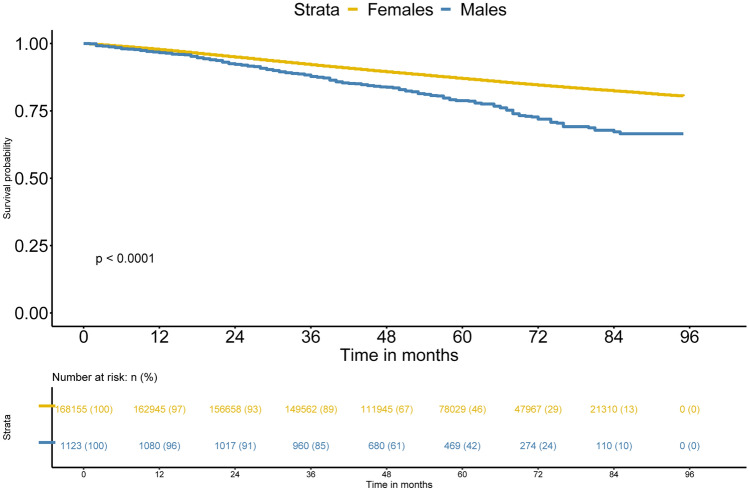
Breast-cancer-specific survival between the MBC and FBC groups.

During the median follow-up of 57 (43–74) months, 116 male patients and 13,140 female patients died from breast cancer. The Kaplan–Meier method showed that the MBC patients had a worse overall survival (OS) than FBC patients. The log-rank test showed a significant difference in the OS between the two groups (log-rank, *p* < 0.001, Fig. 2).

Multivariate Cox regression models were generated to describe the association between clinicopathological characteristics and risk of death. Among FBC patients, results indicated that age (HR = 1.17, 95% CI: 1.16–1.19), black race (HR = 1.24, 95% CI: 1.19–1.30), higher tumor grades (HR_grade2vs. grade1_ = 1.79, 95% CI: 1.65–1.93; HR_grade3vs. grade1_ = 2.98, 95% CI: 2.75–3.23; HR_grade4vs. grade1_ = 3.01, 95% CI: 2.42–3.73), larger tumor size (HR_T2vs.T1_ = 2.34, 95% CI: 2.23–2.45; HR_T3vs.T1_ = 3.46 95% CI: 3.25–3.68; HR_T4vs.T1_ = 3.81, 95% CI: 3.56–4.08), higher lymph node involvement (HR_N1vs.N0_ = 1.84, 95% CI: 1.76–1.92; HR_N2vs.N0_ = 3.06, 95% CI: 2.89–3.24; HR_N3vs.N0_ = 3.57, 95% CI: 3.36–3.80), distant metastasis (HR = 3.95, 95% CI: 3.74–4.17), and type of histology (HR _lobular vs. ductal_ = 1.10, 95% CI: 1.03–1.18) were associated with a higher risk of cancer-related death. On the contrary, ER positive (HR = 0.67, 95% CI: 0.64–0.71), PR positive (HR = 0.61, 95% CI: 0.58–0.64), HER-2 positivity (HR = 0.56, 95% CI: 0.54–0.59), and breast surgery (HR = 0.33, 95% CI: 0.31–0.34) were associated with significantly reduced risks of cancer-related death (Table 2). Similar results were obtained among MBC patients; however, it was noteworthy that PR (HR = 0.68, 95% CI: 0.35–1.31) and HER-2 status (HR = 0.95, 95% CI: 0.55–1.63) did not appear to independently influence cancer-related survival (Table 2). Tumor location was an independent prognostic factor for both MBC and FBC patients. Medial location tumor was associated with a poorer prognosis compared with central and lateral location tumor (HR _central vs. medial_ = 0.95, 95% CI: 0.92–0.98, HR _lateral vs. medial_ = 0.93, 95% CI: 0.90–0.97 in females; HR _central vs. medial_ = 0.44, 95% CI: 0.21–0.94, HR _lateral vs. medial_ = 0.47, 95% CI: 0.24–0.72 in males).

**Table 2:** Multivariate analysis for breast-cancer specific survival stratified by sex in Cox regressions.

	Men		Women	
	HRs (95% CI)	*p*-value	HRs (95% CI)	*p*-value
Age (every10 years)	1.11(1.07–1.16)	0.012	1.17(1.16–1.19)	< 0.001
**Races**				
White	Ref		Ref	
Black	1.59(1.12–2.07)	< 0.001	1.24(1.19–1.30)	< 0.001
Others	0.33 (0.10–1.10)	0.070	0.76(0.71–0.82)	< 0.001
**Grades**				
I (Well)	Ref		Ref	
II (Moderately)	1.49(0.53–4.24)	0.453	1.79(1.65–1.93)	< 0.001
III (Poorly)	2.93(1.04–8.28)	0.042	2.98(2.75–3.23)	< 0.001
IV (Undifferentiated)	16.07(1.55–166.67)	0.020	3.01(2.42–3.73)	< 0.001
**T stages**				
T1	Ref		Ref	
T2	1.84(1.11–3.07)	0.019	2.34(2.23–2.45)	< 0.001
T3	2.45(1.07–5.63)	0.034	3.46(3.25–3.68)	< 0.001
T4	2.25(1.08–4.68)	0.030	3.81(3.56–4.08)	< 0.001
**N stages**				
N0	Ref		Ref	
N1	1.90(1.17–3.08)	0.009	1.84(1.76–1.92)	< 0.001
N2	3.10(1.74–5.52)	< 0.001	3.06(2.89–3.24)	< 0.001
N3	3.32(1.59–6.95)	0.001	3.57(3.36–3.80)	< 0.001
**M stages**				
M0	Ref		Ref	
M1	5.17(2.86–9.34)	< 0.001	3.95(3.74–4.17)	< 0.001
**ER**				
Negative	Ref		Ref	
Positive	0.34(0.14–0.85)	0.021	0.67(0.64–0.71)	< 0.001
**PR**				
Negative	Ref		Ref	
Positive	0.68(0.35–1.31)	0.249	0.61(0.58–0.64)	< 0.001
**HER-2**				
Negative	Ref		Ref	
Positive	0.95(0.55–1.63)	0.845	0.56(0.54–0.59)	< 0.001
Equivocal	1.58(0.97–2.59)	0.067	1.01(0.91–1.13)	0.845
**Surgery**				
No	Ref		Ref	
Yes	0.39(0.19–0.76)	0.006	0.33(0.31–0.34)	< 0.001
**Histology**				
Ductal	Ref		Ref	
Lobular	1.18(0.14–9.74)	0.878	1.10(1.03–1.18)	0.005
Others	0.58(0.27–1.28)	0.179	0.99(0.95–1.05)	0.949
**Tumor locations**				
Medial	Ref		Ref	
Central	0.44(0.21–0.94)	0.035	0.95(0.92–0.98)	0.041
Lateral	0.47(0.24–0.72)	0.021	0.93(0.90–0.97)	0.038
Other	0.58(0.26–1.33)	0.198	1.07(0.99–1.16)	0.062

*ER* estrogen receptor, *PR* progesterone receptor, *HER-2* human epidermal growth factor receptor 2, *HR* hazard ratio, *CI* confidence intervals. The definitions of T, N, M were referred to pathologic stage groups (pTNM).

During the follow up, a total of 9,255 non-breast cancer-related death cases were identified before the occurrence of breast cancer-related death. Tables 3, 4 summarizes adjusted HRs (95%CI) for the association of clinicopathological characteristics with breast cancer related death after taking competing risk events (none breast cancer-related death) into consideration. The associations of clinicopathological characteristics with breast cancer-related death were attenuated but remained significant both in the CS models and the SD models.

**Table 3:** Multivariate analysis for breast-cancer specific survival stratified by sex in the CS models.

	Men		Women	
	HRs (95% CI)	*p*-value	HRs (95% CI)	*p*-value
Age (every10 years)	1.11(1.07–1.16)	0.016	1.17(1.16–1.19)	< 0.001
**Races**				
White	Ref		Ref	
Black	1.59(0.98–2.59)	0.062	1.24(1.19–1.30)	< 0.001
Others	0.33(0.10–1.09)	0.070	0.76(0.71–0.82)	< 0.001
**Grades**				
I (Well)	Ref		Ref	
II (Moderately)	1.49(0.53–4.24)	0.454	1.79(1.65–1.93)	< 0.001
III (Poorly)	2.94(1.04–8.31)	0.042	2.98(2.75–3.23)	< 0.001
IV (Undifferentiated)	16.70(1.61–173.17)	0.018	3.01(2.43–3.74)	< 0.001
**T stages**				
T1	Ref		Ref	
T2	1.84(1.11–3.07)	0.019	2.34(2.23–2.45)	< 0.001
T3	2.46(1.07–5.64)	0.034	3.46(3.25–3.68)	< 0.001
T4	2.27(1.09–4.71)	0.029	3.82(3.57–4.09)	< 0.001
**N stages**				
N0	Ref		Ref	
N1	1.90(1.17–3.08)	0.009	1.84(1.76–1.93)	< 0.001
N2	3.12(1.75–5.55)	< 0.001	3.07(2.90–3.25)	< 0.001
N3	3.34(1.60–6.98)	0.001	3.58(3.37–3.81)	< 0.001
**M stages**				
M0	Ref		Ref	
M1	5.23(2.86–9.34)	< 0.001	3.98(3.78–4.20)	< 0.001
**ER**				
Negative	Ref		Ref	
Positive	0.34(0.14–0.85)	0.020	0.67(0.64–0.70)	< 0.001
**PR**				
Negative	Ref		Ref	
Positive	0.68(0.35–1.31)	0.248	0.61(0.58–0.64)	< 0.001
**HER-2**				
Negative	Ref		Ref	
Positive	0.95(0.55–1.62)	0.838	0.56(0.54–0.59)	< 0.001
Equivocal	1.57(0.98–2.58)	0.088	1.01(0.91–1.13)	0.842
**Surgery**				
No	Ref		Ref	
Yes	0.39(0.20–0.77)	0.007	0.33(0.31–0.34)	< 0.001
**Histology**				
Ductal	Ref		Ref	
Lobular	1.18(0.14–9.75)	0.879	1.10(1.03–1.18)	0.005
Others	0.58(0.27–1.28)	0.180	0.99(0.95–1.05)	0.965
**Tumor locations**				
Medial	Ref		Ref	
Central	0.44(0.20–0.94)	0.034	0.94(0.91–0.98)	0.033
Lateral	0.46(0.19–0.71)	0.019	0.93(0.89–0.96)	0.030
Other	0.58(0.26–1.32)	0.195	1.05(0.97–1.15)	0.112

*ER* estrogen receptor, *PR* progesterone receptor, *HER-2* human epidermal growth factor receptor 2, *HR* hazard ratio, *CI* confidence intervals, *CS model* cause-specific hazard model. The definitions of T, N, M were referred to pathologic stage groups (pTNM).

**Table 4:** Multivariate analysis for breast-cancer specific survival stratified by sex in the SD models.

	Men		Women	
	HR (95%CI)	*p*-value	HR (95%CI)	*p*-value
Age (every10 years)	1.10(1.01–1.36)	0.041	1.14(1.12–1.16)	< 0.001
**Races**				
White	Ref		Ref	
Black	1.47(0.86–2.53)	0.162	1.22(1.16–1.28)	< 0.001
Others	0.33(0.11–1.01)	0.051	0.76(0.71–0.82)	< 0.001
**Grades**				
I (Well)	Ref		Ref	
II (Moderately)	1.41(0.47–4.20)	0.537	1.79(1.66–1.94)	< 0.001
III (Poorly)	2.94(0.97–8.94)	0.057	2.96(2.73–3.21)	< 0.001
IV (Undifferentiated)	14.75(3.73–58.27)	< 0.001	3.01(2.40–3.77)	< 0.001
**T stages**				
T1	Ref		Ref	
T2	1.77(1.05–2.96)	0.031	2.28(2.17–2.40)	< 0.001
T3	2.01(0.81–5.00)	0.132	3.34(3.12–3.57)	< 0.001
T4	1.94(0.86–4.46)	0.113	3.61(3.34–3.92)	< 0.001
**N stages**				
N0	Ref		Ref	
N1	1.81(1.11–2.94)	0.017	1.86(1.78–1.95)	< 0.001
N2	2.90(1.58–5.31)	0.001	3.01(2.82–3.21)	< 0.001
N3	3.40(1.55–7.47)	0.002	3.53(3.28–3.80)	< 0.001
**M stages**				
M0	Ref		Ref	
M1	5.41(2.85–10.25)	< 0.001	3.94(3.70–4.20)	< 0.001
**ER**				
Negative	Ref		Ref	
Positive	0.32(0.12–0.84)	0.020	0.69(0.65–0.73)	< 0.001
**PR**				
Negative	Ref		Ref	
Positive	0.67(0.35–1.28)	0.224	0.61(0.56–0.64)	< 0.001
**HER-2**				
Negative	Ref		Ref	
Positive	0.92(0.51–1.70)	0.794	0.58(0.55–0.61)	< 0.001
Equivocal	1.55(0.93–2.54)	0.133	0.99(0.88–1.11)	0.886
**Surgery**				
No	Ref		Ref	
Yes	0.46(0.23–0.93)	0.031	0.36(0.34–0.38)	< 0.001
**Histology**				
Ductal	Ref		Ref	
Lobular	1.37(0.13–14.05)	0.791	1.11(1.03–1.19)	0.037
Others	0.59(0.27–1.27)	0.178	0.98(0.93–1.04)	0.542
**Tumor locations**				
Medial	Ref		Ref	
Central	0.42(0.20–0.89)	0.024	0.92(0.87–0.96)	0.005
Lateral	0.45(0.22–0.71)	0.019	1.03(0.95–1.12)	0.503
Other	0.65(0.30–1.43)	0.283	0.98(0.92–1.04)	0.427

*ER* estrogen receptor, *PR* progesterone receptor, *HER-2* human epidermal growth factor receptor 2, *HR* hazard ratio, *CI* confidence intervals, *SD model* sub-distribution hazard function model. The definitions of T, N, M were referred to pathologic stage groups (pTNM).

## Discussion

This large-scale population-based study, which makes comparisons between MBC and FBC patients, provides intriguing etiologic and prognostic clues to this disease. Several significant conclusions were made. First, MBC patients have a worse prognosis than FBC patients. Second, there were differences in independent prognostic factors between MBC and FBC patients: PR and HER-2 were independent prognostic factors for FBC but not MBC patients. Finally, breast tumor locations between the two genders were different, which might have an important influence on prognostic results.

In our analysis, MBC patients had a worse overall prognosis than FBC counterparties which was in line with several previous studies. Nahleh et al. found that FBC patients had a significantly longer OS than MBC patients. The median OS for MBC patients was 7.0 years compared with 9.8 years for FBC patients (log-rank test; *p* < 0.05)^9^. Similarly, a study including 2,537 MBC patients also demonstrated that MBC patients had a relatively shorter 5-year survival rate than FBC patients^4^. Several explanations may help to explain this phenomenon. Better prognosis in FBC patients is partly due to the introduction of screening, public awareness, diagnosis at an earlier age with fewer complications, advances in treatment, and standardization of treatment regimens in international guidelines. However, the situation in MBC patients differs a lot compared with female counterparts. First, the breast tissue in men is sparser, and a small tumor would be able to invade the breast skin rapidly. The tumor can also be easily drained into the subareolar lymphatic plexus and thus could lead to a high propensity to metastasize^15^. Second, the prevalence of adjuvant therapies for MBC patients is far behind FBC patients. A recent study using the SEER data from 1996 to 2005 demonstrated that there is a 42% decrease in breast cancer-specific mortality among women compared with only a 28% decrease among men, suggesting that the treatments being used in MBC patients are not as effective as they are for FBC patients^10^. Third, the use of adjuvant therapy in MBC patients is not widespread as FBC patients. In a paper that included 10,173 men with HR-positive breast cancer, men were less likely to receive adjuvant endocrine therapy than women (67.3% vs 78.9%, *p* < 0.001)^16^. Reliable and widespread use of adjuvant chemotherapy and radiotherapy for men is also lacking^17–20^. However, among the patients who were treated with surgery in this current study, male patients were more likely to receive mastectomy than tumorectomy (88.77% vs. 11.23%) when compared to female patients (37.84% vs. 62.16%, *p* < 0.001). The generally higher rate of mastectomies in men could explain why radiotherapy is less often used.

There may be different risk factors between FBC and MBC especially when it is related to PR and HER-2. A population-based study indicated PR status did not appear to independently influence survival among MBC patients^4^. Matthew J’s study also demonstrated PR status did not affect survival in MBC patients^21^. This may be related to the fact that PR status is not a crucial factor for endocrine therapy, and MBC patients are not as sensitive towards endocrine therapy. Little research has focused on HER-2 expression in men. The effectiveness of trastuzumab in HER-2 overexpressing MBC is unproven^22^. In addition, MBC patients with HER-2 overexpression only comprise a small portion of all MBC patients^23,24^, making it difficult to draw a reliable conclusion.

In this current study, the tumor locations between the MBC and FBC patients were markedly different. Among FBC patients, tumors were primarily located in the upper-outer and accounted for 39.16% while other sites in the breast were discovered at lower frequencies, which is in alliance with previous studies^25–28^. This basic observation of asymmetric occurrence of breast cancer has become well accepted but lacks an adequate scientific explanation. A possible explanation is that the upper-outer quadrant of the breast contains a greater proportion of the epithelial tissue, which has a greater chance to occur cancer^29^. In MBC patients, the central position (nipple and central portion) is dominant which accounted for 54.05%. The upper-outer quadrant only made up 15.58%, which was far below the central position. This discrepancy may be caused by the anatomy of the male breast, as there is a larger volume of epithelial breast tissue in the central portion in men^29^. In addition, the prognostic role of the tumor location is also underappreciated, as almost all breast cancer guidelines do not include tumor location as a prognostic factor^30,31^. Yet in our study, tumor location affected the prognosis for both MBC and FBC patients; tumors situated in the medial quadrants of the breast have a worse prognosis compared with those located in lateral quadrants. This finding was compatible with other papers. David K et. al suggested that medial tumor location adversely impacts breast cancer-specific survival and OS in breast cancer patients^32^. Similarly, the Caroline trials indicated that medial location was associated with a 50% excess risk of systemic relapse and breast cancer death compared with lateral tumors^33^.

The poor prognosis of tumors with the internal location may be associated with internal mammary nodes (IMN), which were not conventionally treated. Findings from a previous study have found occult nodal metastases in the internal mammary chain is more likely to be found in tumors with a central or medial location and female breast cancer patients with metastatic axillary nodes^34^. The tumor cell is usually clinically silent in the internal mammary chain, and it might disseminate the disease, especially in node-negative women, who did not receive adjuvant systemic treatment^35^. It has also been observed that 5% of breast cancer patients metastasis to IMNs alone^34^.

Competing risks are common in epidemiological research^35,36^. In the current study, cancer-unrelated death occurred before the identification of cancer-related death. In this competing risk setting, traditional Cox regression may overestimate the absolute risk of cancer-related death, because individuals with a competing (and therefore censored) event are considered to be likely to experience events of interest in the future. The CS model and the SD model are different, and the choice of method should be determined by scientific issues. The CS model may be more suitable for studying the cause of the disease, while the SD model can be used to predict individual risks^14^. Future research should be conducted to explore methodological differences and expand tools to understand competitive risk methods for epidemiological data.

The strength of this current study is the large quantity of data regarding MBC and FBC patients, which allows for a reliable extrapolation of the results. Furthermore, we analyzed the tumor location within the breast, which has been rarely focused on. Moreover, competing risk regressions were further used to validate our results, increasing the accuracy of the study. However, the limitations should also be acknowledged. The main limitation of this study was its missing data, especially on antigens identified by monoclonal antibody Ki-67 status, disease-free survival and adjuvant therapy information. Furthermore, the pathologic information was collected from different hospitals and failed to undergo a centralized review. Lastly, because of the special status of the disease, the number of MBC and FBC patients was very asymmetric. However, the bias was minimized as males and females were studied separately.

## Conclusions

Our retrospective study showed that MBC has a worse overall prognosis than FBC and the independent prognostic factors between MBC and FBC were not entirely the same. In addition, there are vast differences between genders for tumor location, which should be considered by clinicians as a prognostic factor. MBC should be considered as an independent disease. Future research on MBC is needed in many aspects, including molecular pathology, risk factors, genetic contributions diagnostic and therapeutic tools.

## Abbreviations

MBC: Male breast cancer
FBC: Female breast cancer
ER: Estrogen receptor
PR: Progesterone receptor
HER-2: Human epidermal growth factor receptor 2
OS: Overall survival
SEER: Surveillance, epidemiology, and end results
IMN: Internal mammary nodes
CS model: Cause-specific hazard model
SD model: Sub-distribution hazard function model
